# Optimizing Machine Learning-Based Prediction of Terrestrial Dissolved Organic Matter in the Ocean Using Fluorescence and LC-FTMS Data

**DOI:** 10.1021/acsomega.5c02849

**Published:** 2025-07-03

**Authors:** Marlo Bareth, Boris P Koch, Gabriel Zachmann, Xianyu Kong, Oliver J. Lechtenfeld, Sebastian Maneth

**Affiliations:** † Ecological chemistry department, 84597 Alfred-Wegener-Institut Helmholtz-Zentrum für Polar- und Meeresforschung, Am Handelshafen 12, Bremerhaven 27570, Germany; ‡ Faculty 3 - Mathematics and Computer Science, 232610 University of Bremen, Bibliothekstr. 5, Bremen 28359, Germany; § University of applied sciences Bremerhaven, An d. Karlstadt 8, Bremerhaven 27568, Germany; ∥ Department Environmental Analytical Chemistry Research group BioGeoOmics, 28342 Helmholtz Centre for Environmental Research - UFZ, Permoserstr. 15, Leipzig 04318, Germany

## Abstract

Marine dissolved organic matter (DOM) is an extremely complex mixture of organic compounds that plays a crucial role in the global carbon cycle. In the Arctic, climate change accelerates the release of terrestrial organic carbon. Since chemical information is the only way to track DOM sources and fate, it is essential to improve analytical and data science approaches to assess the DOM composition. Here, we compare random forest (RF), support vector machines, and generalized linear models (GLM) to predict a fluorescence-derived proxy for terrestrial DOM based on molecular formula data from liquid chromatography coupled with Fourier transform mass spectrometry (LC-FTMS). We systematically evaluate different data preprocessing, normalization, and ML techniques to optimize prediction accuracy and computational efficiency. Our results show that a generalized linear model (GLM) with sum normalization provides the most accurate and efficient predictions, achieving a normalized root-mean-square error (NRMSE) of 5.7%—close to the precision of the fluorescence measurement. The prediction based on RF regression was slightly less accurate and required significantly more computation time compared to GLM, but it was most robust against data preprocessing and independent of linear correlations. Feature selection significantly improved the performance of all models, with robust predictions obtained using only ca.  2000 of the ca. 70,000 molecular features per sample. Additionally, we assessed the impact of chromatographic retention time on prediction accuracy and explored the key molecular features contributing to terrestrial DOM signatures using Shapley values and permutation importance (for RFs). Our study is a blueprint for the application of ML to enhance the analysis of high-resolution mass spectrometry data, offering a scalable approach for predicting information important for the understanding of marine DOM chemistry.

## Graphical abstract

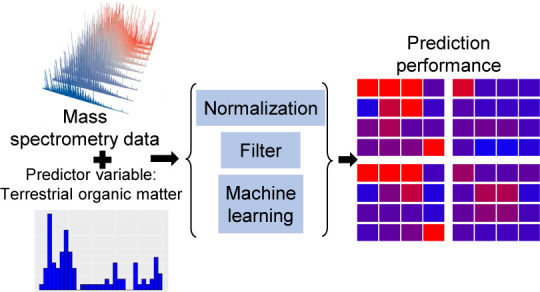

## 1 Introduction

The large reservoir of marine dissolved organic matter (DOM) is an extremely complex mixture of organic compounds.^1,2^ In the dissolved phase, chemical information on individual constituents allows reconstruction of DOM sources, transformations, and their role in the global carbon cycle. The immense chemical complexity that is explored with high-end analytical tools requires new data science approaches to extract relevant chemical information.

DOM results from a plethora of different organic matter sources and biological and chemical alteration processes. It can be categorized into terrestrial (decomposed biomass mainly exported by rivers) and marine origins (mainly marine microalgae). In the context of climate change, understanding and monitoring DOM fluxes in the oceans is crucial for an accurate assessment of the global carbon cycle.^3^ Particularly, the Arctic region is experiencing a larger-than-average increase in temperatures,^4^ resulting in the massive release of terrestrial organic carbon stored in permafrost^5^ that is exported through the Siberian rivers into the Arctic Ocean. The accelerating erosion of the Arctic coastline is another, more direct source of terrestrial DOM.^6^ It is therefore likely that the composition of DOM in the Arctic Ocean will change with yet unknown consequences for the carbon cycle. To track these changes, it is crucial to improve data science approaches to evaluate the enormous amount of data generated by state-of-the-art analytical techniques^7,8^ such as fluorescence spectroscopy and mass spectrometry.

Between 20% and 70% of the DOM can absorb visible and ultraviolet light^9^ and is called chromophoric dissolved organic matter (CDOM). Some of this CDOM emits light as fluorescence (FDOM) when excited with light. Recording a range of intensities of the emission wavelengths for different excitation wavelengths is called excitation–emission matrices (EEMs).^10^ A common approach for evaluating EEMs is parallel factor analysis (PARAFAC),^11,12^ which reduces the multidimensional data into several linear components that can be used for the assessment of DOM sources (e.g., terrestrial input) and biological activity.^13^

Fourier transform ion cyclotron resonance or Orbitrap mass spectrometry (FTMS) can also be used to acquire chemical information about DOM. In FTMS, the DOM components are ionized, and their exact molecular weight and intensity are measured, from which a molecular formula can be calculated. In direct infusion FTMS, a DOM extract is directly ionized, and a single mass spectrum is generated. By coupling reversed-phase liquid chromatography and FTMS (LC-FTMS), the DOM is further separated according to its chemical polarity, so that several mass spectra are recorded along the chromatographic retention time. LC-FTMS has recently been applied to measure the unprocessed filtered seawater.^7^ The main advantage of the new approach is that it avoids bias caused by solid-phase extraction.^14–16^ The resulting LC-FTMS raw data are large (ca. 25 GB per sample), and its efficient evaluation requires modern data analysis methods that are able to detect nonlinear relationships for making predictions using these data.

Several recent studies applied machine learning (ML) for DOM data evaluation, for example, the classification of DOM bioavailability and reactivity based on molecular formula data.^17,18^ Regression tasks have been approached with ML algorithms, such as random forest (RF), gradient boosting, linear regression, and support vector machine, for predicting stable carbon isotope ratios.^16^ LC-FTMS data were used to predict Spearman’s rank correlation coefficients of environmental factors (e.g., land use, sodium, or magnesium concentration) and Shapley Additive Explanation (SHAP) values were calculated to find trends of molecular descriptors.^19^ RF regression was also applied to predict how well molecular formulas correlate with chlorophyll concentration and solar radiation.^20^ The resistance of DOM against UV irradiation was evaluated using multilabel ML regression methods.^21^ Riverine DOM was studied using RFs and SHAP values for key feature analysis and combined model features into chemical groups.^22^

Previous ML-based evaluation of FDOM was typically based on PARAFAC components as features, which were used to predict the origins of pollutants^23,24^ and reduction in oxidant exposure.^25^ Since it is now possible to measure original seawater for its DOM composition using LC-FTMS ,^7^ we can predict for the first time PARAFAC components based on nonextracted marine DOM data.

In our study, we aimed to lay the groundwork for analyzing FTMS and LC-FTMS data sets with ML methods by exploring the feasibility and challenges of using molecular formulas directly as features. For this, we used mass spectrometric and fluorescence data that were acquired from samples taken during a unique full-year ship-based campaign in the central Arctic Ocean (MOSAiC; Multidisciplinary drifting Observatory for the Study of Arctic Climate expedition).^26^ We validated different ML methods for their ability to efficiently predict an exemplary environmental parameter (contribution of terrestrial DOM in the Central Arctic) based on molecular formula data acquired by mass spectrometry. In this case study, we tested multiple methods for data preparation, reduction, and normalization: (i) sum and (ii) ubiquitous sum normalization, (iii) normalization by the dissolved organic carbon (DOC) concentration of a sample, and (iv) log ratio transformation. All of these combinations were optimized using hyperparameter tuning via a grid search. Finally, we explored chemical characteristics of the PARAFAC predictor variable.^27^ We regard our methodological study as a basis for similar applications of ML for LC-FTMS data in the future. Our main research questions for our case study are

(1) How well can ML algorithms predict a fluorescence proxy for terrestrial DOM based on LC-FTMS measurements?
(2) Which preprocessing, normalization, and ML method yield the best, most efficient, and most robust predictions?
(3) Does the consideration of the chromatographic retention time in LC-FTMS improve the prediction?
(4) Which features are most important for good predictions?

The best-performing model in our study was a generalized linear model (GLM) with a root-mean-square error that was only 5.7% of the original scale of the terrestrial component. This model also had the fastest running times for training and tuning. RF regression models were least prone to changes in preprocessing and normalization and also covered nonlinear relationships. Fewer features generally led to improved performance in all machine learning approaches, and precise predictions were achieved using only 2000 features instead of the entirety of ca. 70,000 features (molecular formula and retention time combination) per sample of the LC-FTMS data.

## 2 Methods

### 2.1 Origin of the Water Samples

95 water samples were collected during the “Multidisciplinary Drifting Observatory for the Study of Arctic Climate” (MOSAiC) expedition by the research vessel Polarstern, a drift study from October 2019 to July 2020.^26^ The vessel passively drifted with the ice floes from the Amundsen Basin via the western Nansen Basin and Yermak Plateau to the Fram Strait. A second drift period continued from the end position of the first drift, while the third drift started again from the Amundsen bay.^28^ During the three drifts, the water column below the vessel was sampled from the surface to the bottom water.

### 2.2 LC-FTMS Measurements of DOM in Water Samples

Liquid chromatography (LC) was applied before detection with Fourier transform ion cyclotron resonance mass spectrometry (FTMS). The LC (UltiMate 3000RS, Thermo Fischer Scientific, Waltham, USA) was carried out using a reversed-phase column (ACQUITY HSS T3, 1.8 μm, 100 Å, 150 × 3 mm, Waters, Milford, USA) and a water–methanol gradient^7^ so that the chromatographic retention time represented a decreasing polarity of DOM molecules (see Figure 1 top left, going front to back). In FTMS (solariX XR, Bruker Daltonics, Billerica, USA), the molecules were ionized by electrospray ionization (Apollo II, Bruker Daltonics, Billerica, USA, capillary voltage: 4.3 kV) and a unitless intensity for each ion as molecular mass  (in Dalton) per charge  (*m*/*z*) was recorded in a range from *m*/*z* 150–1000. To improve the signal-to-noise ratio, the mass spectra were summed in 1 min retention time segments. For our study, we used mass spectra from ten segments for each sample with start times between 12.3 and 22.3 min by using a custom script in DataAnalysis (Version 6, Bruker). Molecular formulas were assigned using the R tool UltraMassExplorer^8^ as described in Kong et al. (submitted).^51^

**Figure 1:**
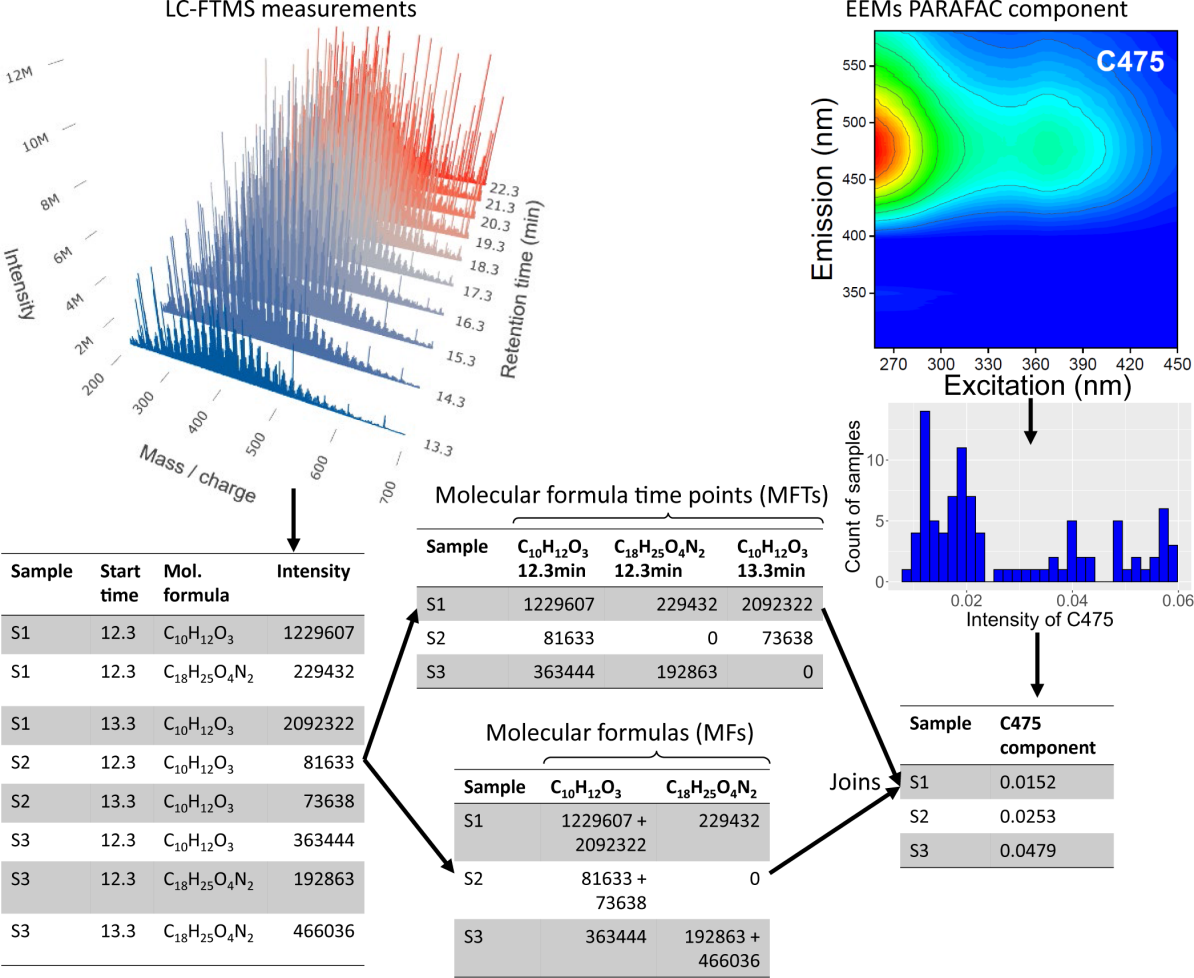
Overview of data preprocessing: LC-FTMS data as mass spectra (top left) and as a table of assigned molecular formulas (bottom left); tables of molecular formula data including the retention time (MFRTs, ″time-aware″; top center) and molecular formulas (MFs) for which the intensity of all retention times were summed (″time agnostic data″; bottom center); the PARAFAC component C475 (right side) was derived from EEM fluorescence analysis (top right) and represents terrestrial dissolved organic matter. Adapted in part (top right EEM) with permission from Kong, 2022.^29^ Copyright 2022 Xianyu Kong (CC BY 4.0).

### 2.3 Fluorescence Data

Three-dimensional excitation–emission matrix spectra (EEMs) were acquired and analyzed (for details cf. Kong et al., 2024 ^28^) using a parallel factor analysis (PARAFAC),^12,30,31^ which is a generalized principal component analysis (PCA).^32^ The PARAFAC component that had its maximum emission at 475 nm was named C475 and was shown to have terrestrial characteristics.^28,33^ It is used as a terrestrial proxy and hence as the predicted variable in our work.

### 2.4 Data Set

The data table for our study consisted of ca. 1.48 million rows, in which the first four columns were the longitude, latitude, depth, and date time. Other columns specified the start time of the retention time segment (each 1 min) from the liquid chromatography, the measured (neutral) mass in Daltons (Da), the corresponding molecular formula of the mass,^34^ the measured mass peak intensity, the intensity of the PARAFAC component (ranging from 0.00927 to 0.0624; Figure 1), and the dissolved organic carbon (DOC) concentration in μmol carbon per kilogram water (μmol/kg; used for DOC normalization).

### 2.5 Wide Table Format

To apply our machine learning methods, the data table described previously was transformed into a wider format, where each sample was represented by exactly one row, resulting in a table of 95 rows. We distinguish two different wide tables obtained from the original table in two different ways:

(1) the time-agnostic table
(2) the time-aware table.

The time-agnostic table had one column for each different molecular formula of the long table. If a molecular formula appeared in a given sample, then the entry for this molecular formula was the mean of all intensities for that sample and formula. If the molecular formula is not present in this sample, then the entry is set to zero. The time aware table has one column for each combination of molecular formula (MF) and segment retention time (MFRT). The entry is the corresponding intensity if it existed or zero otherwise (Figure 1).

### 2.6 Feature Elimination

Many molecular formulas were only present in a few samples (or retention time segments). If the molecular formula feature was not present, it was assigned an intensity of zero in the two wide tables. These entries accounted for an average of 79.3% of the data, and to check the influence of the zero value contribution, two approaches of feature elimination were applied: (i) elimination of each feature that contained at least one zero value (remaining features were called *ubiquitous*) and (ii) elimination of each feature that contained more than 90% zero values (remaining features were called *no low variance*).

### 2.7 Normalization

Different ranges of values, e.g., in DOC concentration or numbers of MFs, can lead to problems in our comparisons of samples. Data normalization can help overcome such problems. We tested three different normalization methods as well as one transformation:

(1) We expect higher intensities when more DOM is present in the sample, as original seawater was measured without equalizing concentrations. For DOC normalization (DOC-N), each mass peak intensity was divided by the DOC concentration of the sample.
(2) Sum normalization (SUM) tries to balance out higher intensities in some samples. Instead of relying on other measurements (e.g., DOC concentration), we divide each intensity by the sum of all intensities of a spectrum.
(3) Ubiquitous sum normalization (UBISUM) divides each intensity by the summed intensities of those molecular formulas that occur in all samples. This gives a common ground between samples and avoids including possible sample contaminations.
(4) Log ratio transformation (ALR) was calculated by taking the logarithm of the ratio between each intensity and the ubiquitous feature with the lowest variance in intensity over all samples. To avoid taking the logarithm of zero assigned intensities, one-third of the lowest intensity is used as a zero replacement.

The normalizations were applied to the two wide table formats, which were filtered by combinations of feature elimination. The two wide table formats yielded 32 different preprocessing combinations for four feature elimination combinations (none, ubiquitous filtering, low variances excluding filtering, or both) and four normalizations. The C475 component was normalized using the *Z*-score, setting the mean as zero and converting the scaling above or below in units of standard deviations. As the *Z* score takes the difference to the mean C475 value, positive values can be considered to indicate terrestrial influence, while negative normalized C475 values indicate a marine origin, assuming that only marine and terrestrial DOM sources predominate in the Arctic Ocean.

### 2.8 Machine Learning Methods

To train the machine learning models, we took a data set and trained the model to make predictions on the used data. We split the data set into two sets: training (80%) and testing (20%). The former was used to train the model and the latter was used to evaluate the models’ performance on unseen data. In our study, we applied three machine learning methods: (i) generalized linear model (GLM) as a linear approach,^35^ (ii) random forest regression (RF),^36^ and (iii) support vector regression (SVR)^37^ as nonlinear approaches.

GLMs extend linear regression, which gives a prediction by building the sum of the products of a weight (beta values) and the model feature (e.g., the intensity of a molecular formula). One extension is a random component, which is a class of probability distributions that the response is assumed to follow. The other extension is the link function; it describes the relationship between the linear predictor and the prediction mean. We use a modified version of GLMs, which includes a regularization that tries to prevent overfitting by limiting the magnitude of the weights of the linear terms of the GLM. This “elastic net regularization” has two parameters: lambda, the overall strength of the regularization, and alpha, which controls the norm that is used for the regularization by taking a value between zero and one. When alpha is one, it only applies the L1 norm (Manhattan distance) and is known as lasso regression. When comparing two highly correlated features, one weight is reduced to zero. When alpha is zero, the complete regularization is based on the ridge regression, which uses the L2 norm (Euclidean distance) and reduces the predictors but keeps all features.

The GLM method was tested due to its linear approach. Linear regression was previously used in DOM analysis but was outperformed by the nonlinear approaches.^21^ Yet, we included the model, as its simplicity has two useful benefits. The GLMs can be trained quickly and provide simple access to the feature importance metric. To avoid overfitting and improve the performance, we regularized the features with the elastic net.

RF regression models consist of multiple decision trees that form an average of different predictions. For the construction of each tree, only a subset of the data is available to create independent trees. This means not all features and samples are available. The decision tree consists of nodes, where the data are split into two subsets and directed into nodes further down in the tree. The splitting is done by trying numerous split candidates, which is called mtry, where the data is split according to one feature and a threshold. How well a split is considered at dividing the data into subsets is determined by a split rule. The end of the repeated splitting is reached when a minimum number of samples remain in any child node, which is referred to as the min node size. Such leaf nodes are assigned the average of the remaining sample values. RFs were chosen in this study because they can show nonlinear relationships and are resistant to overfitting.^36,38^ RFs were previously used in DOM analysis using mass spectrometry^18,20,21^ and remote sensing data.^39^

SVR models try to find the best fit for the data within a margin of tolerance. This can be visualized in three dimensions as a tube or a long cylinder that is fitted to minimize the error of points outside the tube. The errors inside the tube are not considered. How strictly the error is penalized is defined by the cost parameter. Sigma determines how influential single samples are on the model. To make the model more suitable for different data sets, a kernel can be applied. They map the input into a high-dimensional feature space. This allows the SVM to handle nonlinear data. The kernel functions can be based on different functions like polynomial or radial basis functions. SVMs were chosen in our study as a second nonlinear ML method that was previously applied to DOM^18,21^ and mass spectrometry data.^40,41^ With GLMs already covering linear relationships, we chose the polynomial (SVMPOLY) and radial basis function kernels (SVMRBF) in our test.

### 2.9 Evaluation Metrics

The model performance was measured using the root-mean-square error (RMSE) between the true PARAFAC component intensity and the predicted PARAFAC component intensity across all samples of the test set. With the predicted variable being a unitless PARAFAC component, we decided to convert the RMSE for easier understanding. The normalized RMSE (NRMSE) was calculated by normalizing RMSE by the original scale of C475 values in the data set (see Figure 1, box plot on the right). This means the NRMSE is the RMSE divided by the difference between the maximum and minimum of C475 values. During the training of models on the training set with different hyperparameter combinations (e.g., RFs with varying mtry values), RMSE was used to compare the models. We utilized NRMSE for reporting the results of the model that was best performing during training, based on which the samples from the test set were predicted. To check whether an RMSE was an outlier, we utilized the standard error of the mean, which is the standard deviation divided by the square root of the number of repeated model trainings. For the significance tests of experiment comparisons, we use the two-sided rank test with a continuity correction. A *p*-value of less than 0.05 is considered significant.

After tuning the models, we aimed to find the features that affected the model predictions the most, which we consider to be important. To evaluate these key features in RF models, the SHAP values and permutation importance determined the feature importance. For GLMs, beta values were used, as they weigh the feature in a direction and thus can be considered on a scale of importance. This works only when the intercept of the linear model is zero. This way, a positive weight indicates a terrestrial feature. Sets of these features were compared based on the Jaccard similarity, which measures the similarity between two sets and has a range from zero, for sets without overlaps, to one, for equal sets.

Recursive feature elimination (RFE) is performed to check the model performances with different numbers of features. It is done by constructing a random forest, assessing the importance of the features, and removing the least important features from the data set. Then, a new random forest was built, and the next iteration of RFs was calculated until no features were left. For this, the permutation importance of the out-of-bag error was used. This means that the samples that were not included for the building of the trees were used to evaluate the features by shuffling the feature values between these samples and comparing the corresponding predictions. We used a step size of 10% of the currently remaining features to be removed, giving us a good resolution at small feature numbers but not requiring many models for high feature counts. To make the results more reliable, a cross-validation with 10-fold was performed in each step. Each fold was used once as an evaluation set to avoid over- or underscoring features for a single data split and the model only being able to predict the training data.

#### 2.9.1 SHAP Values

The permutation importance provides insight into how important a feature is to find a good prediction. It does not grant insight into the direction in which a high, or low, value of a feature shifted the prediction. For this, SHAP (Shapley additive explanation) values can be used. The original Shapley values are based on game theory and how different players of a team contributed to a profit. The contribution is calculated by building coalitions of different players and player numbers and then predicting their profit, and then each participants’ contribution can be calculated. This is done for the features of ML predictions as well by using SHAP values. They do simulate numerous possible feature coalitions instead of calculating all coalitions, as the number of coalitions is exponentially increased by the number of features. Adding the SHAP values together results in a difference from the mean predicted value. The drawback of SHAP values is that they are calculated for each sample and therefore are different for a feature when comparing different samples.^42^

### 2.10 Experimental Setup

The programming language R (version: 4.3.1)^43^ was used for implementation, as it allows seamless interaction with the UltraMassExplorer package.^8^ The models were constructed by using the caret package. Our experiments started with splitting the data tables into the commonly used 80% training set and 20% test set by using the *createDataPartition* method from the caret, which is designed to build similar sets based on the C475 intensity. This avoided bias in the distribution of C475 intensities in each set, but due to computational limitations, a nested cross-validation where the test/training split would have been segmented as well was not feasible. The complete model training was only performed on the training set, and we used a grid search with 10-fold cross-validation and ten repeats. The grid search builds models with different combinations of hyperparameters that influence the models. After initial tests, we selected some hyperparameters to have fewer values in the tuning grid to reduce the computation time of the training process. The 10-fold cross-validation split the training set into ten parts, called folds. Nine of the folds were used to train the model, and the other part was used to validate the model performance. For each fold, this was performed for a total of ten iterations. Subsequently, the best-performing models were selected and evaluated based on the test set. This test set was never used in training and was used to check if the model only performed well on the training data and not on new data (overfitting). We calculated the RMSE and NRMSE based on the test set evaluation, to detect overfitting. For a few experiments, we repeated the cross-validation with different random seeds to estimate the impact of the training and test data split.

For GLM, we utilized the glmnet package (version 4.1-8). We assumed the error to be normally distributed and used the linear link function. The grid search contained the two parameters of elastic nets: alpha and lambda. alpha was increased in 0.05 increments during the hyperparameter tuning. lambda took values from 0 to 2 in 0.05 increments. As mentioned in the previous chapter, we use an intercept of zero.

For RF, the ranger method from caret was used. 1000 trees per random forest were found to be an acceptable compromise between performance and time required based on initial tests. The split rule was left as the variance for all experiments. Values for mtry were tested in one-sixth increments of the number of features, up to the whole. The minimum node size was tested for values of 3, 5, 7, 10, and 15, having bigger steps between the higher values. As RF does not yield the same result every time a model is trained, 500 RFs were built after the hyperparameter tuning with the selected optimal tuning parameters.

For the polynomial kernel SVR, we tested cost hyperparameters of 0.01, 0.1, 0.25, 0.5, 0.75, and 1, as well as polynomial degrees from one to five degrees at a fixed scale parameter of one. The grid for the radial basis function kernel was built from the cost and sigma values. Cost parameter values of 0.001, 0.01, 0.1, 0.25, 0.5, 0.75, 0.9, 0.99, and 1 were used. We evaluated sigma values ranging from 10^–6^ to 1, including 10^–6^ up to 10^–5^ in factor 2.5 increments, 2 × 10^–5^, a coarser grid of 5 × 10^–5^ up to 5 × 10^–4^ in factor 5 increments, and a sparse upper scale of 10^–3^ increasing by a factor of 10, up to one.

For selected models, we also checked if the NRMSE was an outlier. For this, we repeated the tuning 1000 times and used different splits for the training and test sets.

The model tuning and repeated model trainings were run on a server node that had 256 GB RAM and two AMD Rome Epyc 7702 processors with 128 cores in total (albedo server, AWI). We used 120 threads in the experiments to allow background processes to execute. All other preprocessing and experiments were conducted on a workstation with an Intel i7-1165G7 processor, an Nvidia T500 graphics card, and 32 GB RAM.

## 3 Results

The preprocessing of the molecular formula data resulted in 32 data tables, each of which was used to train four ML methods, leading to 64 models without (Figure 2) and 64 models with (Figure 3) low variance filtering.

**Figure 2:**
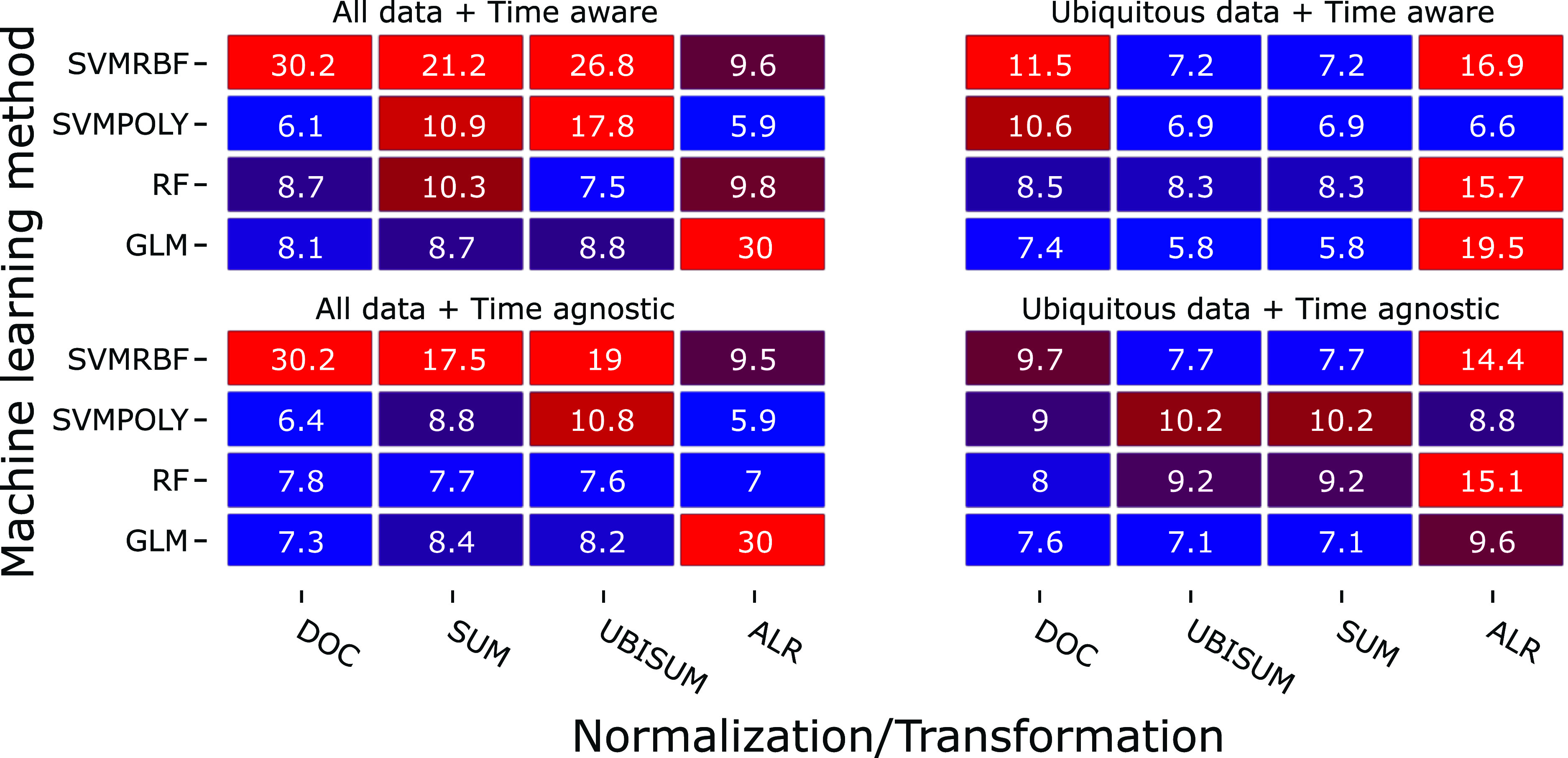
Model performance based on all molecular formula features: Normalized root-mean-square errors (NRMSE) are expressed in percent of the original scale of C475.

**Figure 3:**
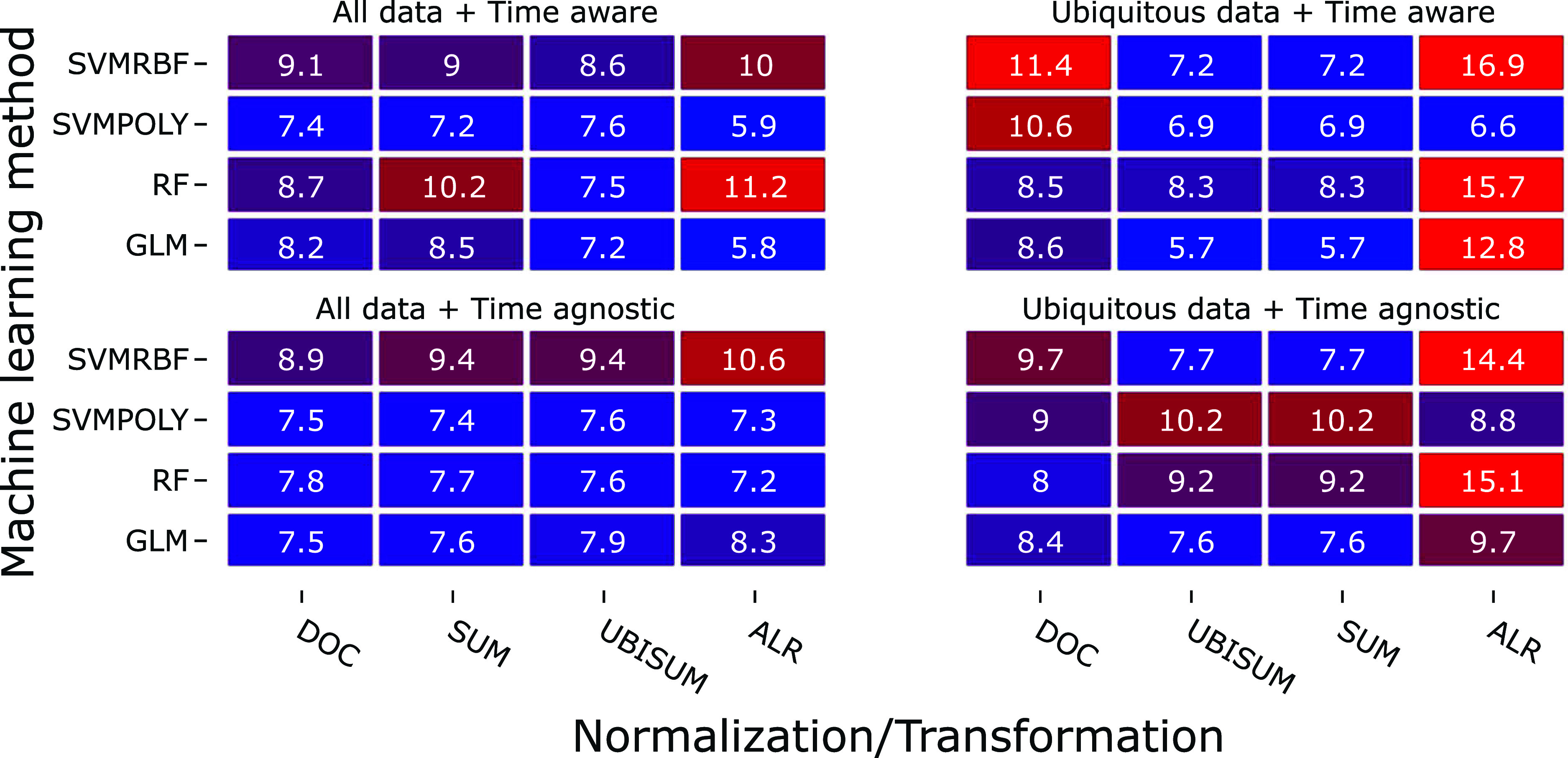
Model performance after the removal of low variance features: normalized root-mean-square errors (NRMSE) in percent of the original scale of C475.

### 3.1 Prediction Performance Using All Features

When comparing the NRMSE values, the best prediction was achieved with a GLM using ubiquitous data, the retention time dimension, and SUM or UBISUM normalization (Figure 2, top right). GLMs performed best or second best (13 out of 16 variants), except for the models using ALR-transformed data. The SVM models performed worst when using all data, with the exception of the models that used ALR-transformed data. GLM was the only method that consistently yielded an NRMSE below 10% when using ALR transformation. RF performance was best for the models that included all time-aware data using SUM and UBISUM normalization (lower left subplot; Figure 2), whereas in the ubiquitous data and time-aware experiments (top right subplot; Figure 2), RF had the highest error. Generally, the NRMSE was similar or smaller when using only the ubiquitous data, compared to the use of all data. The performance of models based on time-aware data (top of Figure 2) did not show significant differences (*p* = 0.144) compared to experiments with time-agnostic data (bottom of Figure 2). It should be noted that for ubiquitous data, the sum of intensities (SUM) was the same as the sum of ubiquitous features (UBISUM), leading to identical errors for models except RFs. The other metrics, such as *R* ^2^ and mean absolute error, showed similar trends for all entries (Figures S6– S8), but RFs did have the highest or second-highest *R* ^2^ in 11 out of 16 cases. For the high error occurring in ALR-transformed GLMs of unfiltered, time-aware data, we exemplarily performed 1000 repeated model trainings of the hyperparameter search with different train-test splits. The STEM was 0.547% NRMSE for 100 repeated splits and 0.184% NRMSE for 1000 repeated splits. For quality control, the repeated cross-validation was performed using the unfiltered time-aware data with DOC normalization that was used to train a GLM (Figure 2 top left; bottom row, first column). It was selected because the model had the highest feature count possible and otherwise the best-performing normalization. The models showed an average NRMSE over 1000 models trained of 8.87%, compared to 8.1% in the heatmap.

### 3.2 Prediction Performance after the Removal of Low Variance Features

Removing the low-variance features led to a 61–62% reduction in the number of features and a significantly (*p* = 0.0417) better performance (Figure 3).

In contrast to all data experiments (Figure 2), the removal of low-variance features overall led to improved performance. The NRMSE values were consistently below 10%, except for five experiments, when excluding ALR transformation. A significant change in NRMSE was found over all experiments (*p* = 0.0417) when using the ALR-transformed models. The highest NRMSE with 16.9% is around half as high as the worst case in the nonfiltered experiments (30.2%). However, no significant (*p* = 0.702) difference was found between the ubiquitous filtered data without (Figure 2 right) and with low-variance filtering (Figure 3 right). The difference between model performance using only low-variance filtered data (Figure 3 left) and those with additionally ubiquitous filtered data (Figure 3 right) was not significant when ALR transformation was excluded (*p* = 0.643). A paired Wilcoxon signed rank test with continuity correction between the time-aware and time-agnostic models found no significant difference (*p* = 0.417) between the two pairs of 64 models and also no significant difference between the low-variance filtered data (*p* = 0.660). GLMs based on the time-aware and SUM/UBISUM normalization (Figure 3 top right) yielded the best predictions, similar to the results of the low variance including data sets (Figure 2). SVM with a polynomial kernel performed best or second-best in most (seven out of eight) preprocessings for data, which were not further filtered by ubiquitous contents (Figure 3 left). GLMs performed equally well in 14 out of 16 experiments. SVM with a polynomial kernel outperformed GLM or performed equal in half of the 16 experiments, where five out of these eight used time-agnostic data. Similar trends were observed for the *R* ^2^ and mean absolute error (see Figures S7 and S9).

### 3.3 Random Forest Regression

Although the RF models did not yield the overall best performance when focusing on NRMSE, the RFs did have high *R* ^2^ values, did not require linear relationships, and performed well across all tested normalization (excluding ALR transformation) and filtering approaches (Figures 2 and 3). Since RF regression was the only method that was not deterministic, we assessed the prediction error introduced by randomness. The performances of 500 repeated runs were evaluated for RFs between variants that were not filtered to exclude low variances. The NRMSE introduced by RF uncertainty was much smaller than the errors between the models (details in Figure S1), and the RF uncertainty was significantly lower than the NRMSE differences between time-aware and time-agnostic models (*p* = 0.0078).

#### 3.3.1 Recursive Feature Elimination: Evaluation of Random Forest Model Performance

We tested recursive feature elimination to validate how the model’s performance changes with the number of features used in each model, where we used the complete time-aware data with UBISUM normalized. The importance is based on the permutation importance obtained from the out-of-bag error that is accessible in RF models from the caret and ranger.

In each iteration of the recursive feature elimination, the 10% least important features were removed (Figure 4) and a 10-fold cross-validation and recalculation of the importance were performed. For less than ca. 2000 features, the error increased from 10% to about 12% NRMSE for less than 300 features. A plateau of about 30% NRMSE was reached at 20 or fewer features.

**Figure 4:**
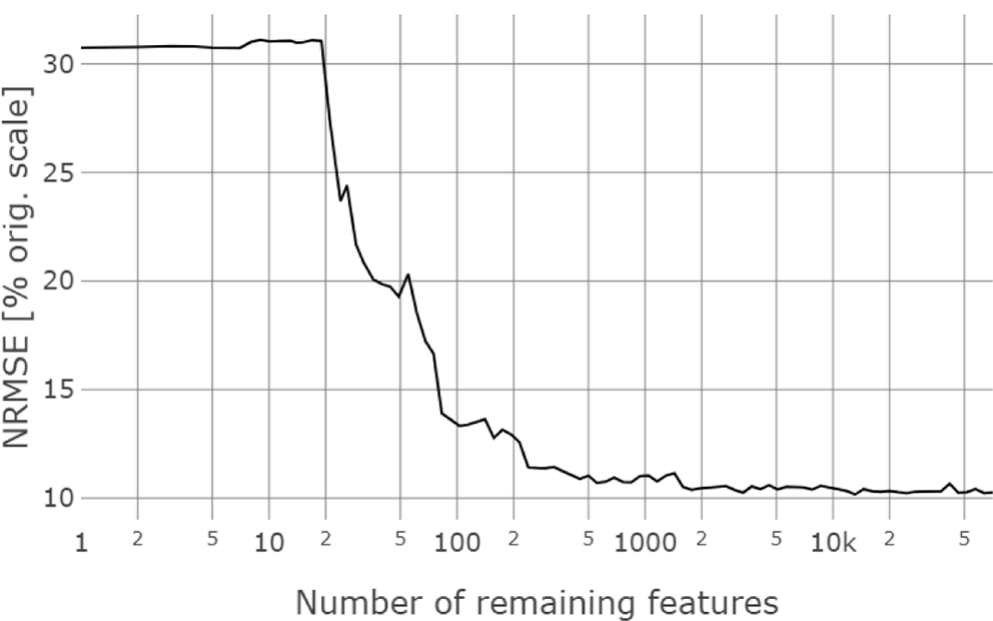
Recursive feature elimination: normalized root-mean-square error (NRMSE) of the random forest experiment based on time-aware DOC normalized and nonfiltered data; the initial number of features is *n* = 70,683.

### 3.4 Feature Importances

As a first step for key feature analysis, we used RF permutation importance and SHAP values to identify the most important molecular formulas and MFRTs, respectively, that represented the C475 fluorescence component.

#### 3.4.1 Random Forest Permutation Importance

The permutation importance of RFs is a commonly applied parameter to identify the most relevant features. To check how reliable the found importance values were, we created 500 forests. The hyperparameter optimization was performed only once at the beginning and then used for RF creation. From these, we selected a number of top-ranking features from each forest, increasing in number (1 to 100). Then, we built a set that contained all unique formulas that were included in these 500 forests. The RFs reached 592 different MFRTs in the top 100-ranked important features by permutation importance, using a nonfiltered data set, among which ALR transformation resulted in the best model (see Figure S2). If time-agnostic data were used, the number of different features reached around 300 MFs. Similar numbers of varying features were observed from ranking the SHAP values of the built RFs (see Figure S3 for time-aware data and Figure S4 for time-agnostic data).

#### 3.4.2 SHAP Values of Random Forest Models

To identify the importance of the features in the RFs, we utilized SHAP values. We associated positive SHAP values with terrestrial features as our prediction variable was high for terrestrial samples. Therefore, negative SHAP values were assumed to represent marine features. SHAP values for the features were calculated on a sample-wise basis and were calculated from the RF model that excluded low-variance features and used DOC-N and time-agnostic data. To get an overview of different samples, we selected three samples from the test set. We selected one sample that was close to the average C475 of the test set as well as the most terrestrial and marine samples. To evaluate the composition of the features, we scaled the positive SHAP values from one to zero and computed a weighted average composition for these samples. For the average terrestrial sample, the positive SHAP values accumulated in the lower mass region of 200–400 *m*/*z*. When scaling these SHAP values to between zero and one and multiplying them with the composition of the feature, the average composition is C_18.1_H_21.5_N_1_O_9.6_S_0.18_. This means a sulfur-to-carbon atom (S/C) ratio of 0.009 and a carbon-to-nitrogen atom (N/C) ratio of 0.06. The very marine-sided sample had a composition of C_18.3_H_24_N_1.4_O_10_S_0.24_. The S/C ratio was 0.013, and the N/C ratio was 0.08. For the very terrestrial sample, the composition of a MF with positive SHAP values was C_18.2_H_21.1_N_1_O_9.6_S_0.18_, yielding a S/C ratio of 0.01 and a N/C ratio of 0.05, which were similar to the average terrestrial sample.

From the average terrestrial sample from the test set, we found 3546 positive SHAP values, which indicated terrestrial content. A previous analysis of this data, via linear regression, found 1450 MFs to be terrestrial.^28,29^ The overlap of the two sets was 759 MFs, and the Jaccard similarity between the sets was 0.18. To compare the permutation importance as well, we used the 1000 repeated RF runs with DOC normalization, where we selected the top 100 features by permutation importance. Around 592 unique MFRTs were identified in the time-aware data. For time-agnostic data, the number is 316 MFs. The latter was compared to the 1450 terrestrial features^28,29^ and yielded a 0.16 Jaccard similarity between the sets.

Lastly, we compare our sets from SHAP values and from permutation importance to each other. We found a Jaccard similarity of 0.70 for the time-aware data (MFRTs) and of 0.74 for the time-agnostic data (MFs).

### 3.5 Running Times

The computation time for the grid search was strongly dependent on the machine learning methods (Figure 5). GLMs were quickest for each preprocessing combination, whereas the RF took the longest time to compute, especially when no feature reduction and time-aware data were used. In this case, the hyperparameter search for the Random forest took close to 200 min, with only small variations between the normalization types (191 min minimum and 201 min maximum).

**Figure 5:**
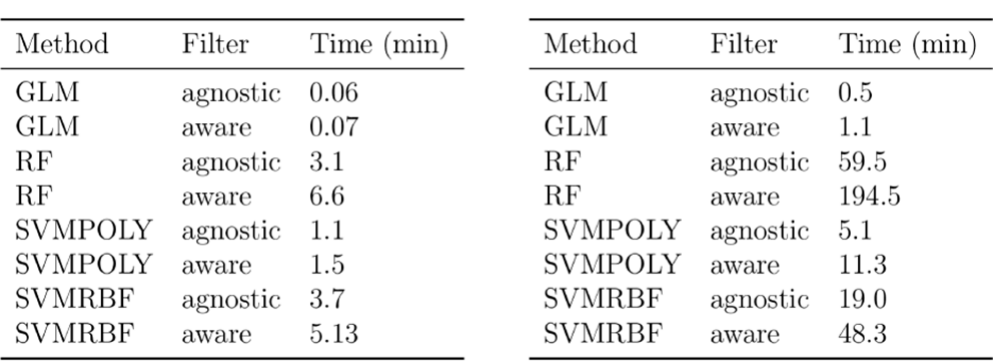
Running times for the grid search averaged over all different normalizations for ubiquitous data (left) and all data (right).

It is important to note that our experiments were executed on a high-performance computer employing 120 cores running in parallel (exemplary models trained on the laptop took 15–20 times longer). For ubiquitous data, GLMs were faster than RFs by a factor of between 53 (time-agnostic) and 91 (time-aware) times, and when we compare GLMs to SVMPOLY, then they are still faster by a factor of about 19 (Figure 5). When considering all data, GLMs are faster than RFs by factors between 125 and 170 and GLMs are faster than SVMPOLY by a factor of around 10.

## 4 Discussion

### 4.1 How Well Can ML Algorithms Predict the Fluorescence Proxy for Terrestrial DOM Based on LC-FTMS Measurements?

The most accurate, molecular-formula-based prediction for the fluorescence component C475, a proxy for terrestrial organic matter, was achieved from the GLM. Depending on the choice of feature type (molecular formulas or molecular formula time points) and preprocessing, the lowest prediction error showed an NRMSE of 5.7% of the original range of C475. This value was only slightly above the precision of the fluorescence method itself (below a factor of 2), which we estimated to have an NRMSE of 3.5% of the total range of intensities for all samples. This NRMSE estimation was calculated based on the repeatability of C475 values in the deep Arctic Ocean (400 m or below), which generally showed very similar fluorescence signals (cf. Kong et al. 2024). In a previous study, five ML models were tested for their ability to predict the stable carbon isotope ratio δ^13^C from molecular formula data.^16^ Based on solid-phase extracted DOM, the linear SVM kernel achieved the best predictions, in contrast to our study, where GLMs and RF regression showed the best performance. The authors report a prediction error for δ^13^C of 0.3‰. To make this value comparable to our results, we scaled this error to the entire range of δ^13^C values (−27.7 to −21.9‰). This resulted in an NRMSE of 5.1%, which is comparable to our best value of 5.7% for the GLM prediction of terrestrial fluorescence. Our best-performing SVM (NRMSE of 5.9%) used a polynomial kernel of first degree and was comparable to the results by Yi et al.^16^ who used a linear SVM kernel.

### 4.2 Which Combination of Preprocessing, Normalization, and ML Method Yields the Best Predictions?

Generally, the best predictions for C475 were achieved with data that were filtered for ubiquitous formulas and normalized by SUM (equivalent to UBISUM normalization). The sum normalization was also used by Yi et al.,^16^ but the molecular formula assignment differed slightly: While we validated and filtered the molecular formula set by limiting the double-bond equivalents minus oxygen (DBE-O^44^) to a maximum of ten, Yi et al. (2003) filtered formulas by H/C < 3 and O/C < 1.5.^16^ As expected, the choice of preprocessing and the type of machine learning model had a large influence on the performance of the different models. Somewhat surprisingly, a decrease in the number of features led to an improved prediction. The performance of most models was improved by removing low-variance features. Alternatively, filtering for ubiquitous formulas was performed if the low-variance filtering was not applied. The only exceptions were the RF experiments, which delivered robust performance irrespective of the number of features when excluding the ALR-transformed data.

The different normalization methods did not influence the model performance substantially. The ALR transformation prevented high error rates for the polynomial kernel SVMs, when unfiltered data were used, but increased the error for all other combinations. We discuss this in a later paragraph.

The time-aware data that included only the ubiquitous features and normalized by the sum of intensities of each segment performed best, with an NRMSE of 5.7% for GLMs. This was nearly (0.1% NRMSE) independent of low variance filtering, which could be due to the ubiquitous filter and excluding low variance features, since this way every sample measured a signal and the low variance features mostly are those with many cases of zero filling.

We clearly found that some model setups were particularly sensitive to higher numbers of features in combination with the low number of samples (e.g., SVMs with RBF kernels, Figure 2). RF regression and GLMs did handle the unfiltered data consistently well for most cases (Figures 2 and 3). We assumed that RFs cope better with a relatively large number of features, as the large number of split candidates for each tree filters out the low-variance features as bad splits.^38,45^ Several experiments in our study yielded prediction performances with high errors (NRMSE of greater than 10%). The maximum NRMSE of 30.2% is a factor of more than eight above the precision of the fluorescence measurement. High uncertainty was observed particularly for those experiments that were based on a large number of features (molecular formula time points and no removal of low-variance features; Figure 2). We suspect that the situational large prediction errors for SVMs were caused by failing to find a solution to the equation system because too few samples per feature.

We hypothesize that the high test set errors of ALR-transformed models were caused by overfitting, meaning that the model adjusted too much to the trained data and does not generalize well. Evidence for that is that the model has high errors on the test set while showing low training set errors, where the RMSE was up to three times as high in the test data evaluation as in the chosen model from the grid search. Our theory for the overfitting was induced by collinearities arising from the original data. They could be a coincidence or induced by the ALR transformation. We have no other hypothesis about why the overfitting only affected ALR models to such a degree.

One general problem could also be the splitting of the test set. With only 95 samples, the left-out samples possibly were not well-balanced for the features with high weights that were found by the model. This would explain why this behavior was not seen in the low-variance filtered set with no ubiquitous filtering. Similar problems with a small test set have been observed and were approached via a pooled test set, which could be an approach for our future work.^46^

In our study, the reduction of the number of features led to an improved prediction performance for most experiments and a faster processing time, particularly for RFs. Three processes reduced the number of features: (i) creating time-agnostic data from the original retention time data (molecular formula time points, MFRTs), (ii) filtering for formulas or MFRTs that occurred in all samples (ubiquitous), and (iii) removing features with low variance. Utilizing all available features led to a larger variability of the prediction performance and increased the influence of the normalization method (see Figure 2). Applying low-variance feature filtering (ca. 61%) minimized the differences in performance between the models. It also improved the performance of the models with previously situational high errors like SVMs and the GLMs with ALR transformation in cases where ubiquitous filtering was not applied (Figures 2 and 3 left). It also reduced the time for tuning the RF and SVMRBF models by circa three times (see Figure 5). Using only ubiquitous features, all normalizations except ALR normalization performed well over most experiments and reduced the calculation time from unfiltered data by 5–15 times (see Figure 5). The reduction of features is generally beneficial, as each feature adds a new dimension that needs data to cover it.^47^ The exclusion of features allows the data to fill more locations in the reduced dimensional space.^48^ Only utilizing the ubiquitous features, the feature counts may reach areas of too-strong filtering, where information was lost. This was supported by the RFE, where NRMSE rose quickly for data sets below 2000 features. The time-agnostic, ubiquitous data set had only ca. 1800 features, which may explain the increased error.

In our study, the number of samples is small relative to the number of features. Therefore, some features are likely to be highly correlated. Using RFE, we found that ca. 2000 out of ca. 70,000 features were sufficient to create an RF model with comparable prediction power. The filtering here was only based on the feature importance, which shows the value of preprocessing by excluding low-variance features or keeping only ubiquitous features to avoid unnecessary computational effort. The results from the RFE also relied on cross-validation to protect the feature selection and model performance from overfitting. The reported error is based on the omitted folds and not on a separately kept test set. As the model performed similar to the other RFs with filtered data, we do not see proof of overfitting in the RFE. We found a plateau in the required minimum number of features, an observation that was also seen in previous studies using RFE and random forests.^49^ Our results using RFE agreed with the low NRMSE after low-variance filtering. These findings led us to advise using at least one form of filtering to counteract high dimensionality. We did not analyze whether the small number of features required is based on the sparse abundance or if a lack of chemical relevance of molecular formula caused this. This will be the subject of a subsequent study.

### 4.3 Does the Consideration of the Chromatographic Retention Time Improve the Prediction?

In standard liquid chromatography, time-dependent data assume that the time windows are independent of each other; i.e., the same molecular formula present at different retention times is considered two independent features. Our comparison of time-agnostic and time-aware experiments made it possible to validate the significance of chromatographic retention time. Time-aware features that included retention time improved the GLM performance (by around 0.24% NRMSE) but decreased the performance in most RF experiments. Overall, there was no statistical difference found between the prediction performance based on the time-awareness or time-agnostic data, although the retention time represents chemical information (polarity of the molecules) and allows a better separation of the complex organic material. When removing the time dimension, we traded better separation of compounds for an input matrix with less sparsity and an experimental setup with wider availability in the community. The adaptability of RFs to sparse data^38^ could have reduced the observable differences between both data sets (time-agnostic and aware), but both were sparse. Still, predictions of RFs using time-agnostic data were better, which we attribute to less overfitting due to the increased variance per feature and the general reduction in feature dimensions.

The signals of one structural formula have an elution window of around 30 s and a bell-shaped intensity curve. We thus assume the majority of each structural formula to be in one retention time window, even though this is a simplification. Other approaches avoid this by selecting specific retention time windows with gaps between them.^19^

### 4.4 Which Key Features are Most Important for a Good Prediction?

Computing SHAP values and permutation importance was suitable to isolate the important features in the complex mass spectrometry data set. Our findings are particularly helpful for targeted analytical approaches that aim to identify structural information from the key features identified. Terrestrial MFs were characterized by lower masses compared to marine MFs, which matched the results of previous studies.^28,50^ The average terrestrial N/C element ratio in previous work (Kong et al., submitted^51^) was lower than 0.001, in contrast to our results that showed an average N/C of 0.06 for terrestrial MFs identified by SHAP values in a sample with high terrestrial DOM contribution and an average N/C of 0.08 for a sample with predominately marine DOM contribution. For S/C ratios, a similar trend was observed.

The different ratios indicated vastly different molecular formulas compared to previous work (Kong et al., submitted^51^), even though the N/C and S/C ratios were consistently lower in terrestrial compared to marine DOM in both studies. If we compare the number of identified top 100 important features in 1000 runs, the chance of a feature appearing in a model’s top 100 features is low (less than 16%, ca. 600 unique features in the top 100 features). When we used time-agnostic data, the number of identical, important features between models increased to 30%. One possible explanation is that time-aware data can predict fluorescence well on each time slice but with different MFRTs. Another explanation is the reduced feature space. With roughly one-third of the features in the time-agnostic data (23,835 compared to 70,683, around 33.7%), only around one-third of the features are found in the 1000 repeated RF runs, indicating a similar spread of total features being deemed important. The similar trends between the permutation importance features and the SHAP value features were only partially supported, as they had a Jaccard similarity of 0.7 between the ca. 600 features of the top 100 ALR time-aware data. The time-agnostic top features were even more similar to a Jaccard similarity of 0.74. Previously reported stable groups of similar features from rivers^52^ were not discovered by our key feature search. Reasons for this are likely the longer distance in the ocean, as the samples from the MOSAiC cruise were not taken from the rivers directly but mostly from the Central Arctic Ocean. This likely allowed degradation of the MFs by degrading ultraviolet radiation^53^ or microorganisms. The cruise was performed over a complete year, and seasons were not a parameter we considered in our analysis, but changes in DOM were shown to be more dependent on region than on the season.^28^

All found key features only covered a small fraction of DOM. The direct measurement of ocean water with LC-FTMS reduced methodological bias of identified DOM but our models were also dependent on the PARAFAC component from the EEMs. As these EEMs measure the fluorescence, all found features and all trained models were blind to DOM that was not part of the small subset of fluorescent DOM. In a new study, C475 was correlated to δ^13^C and salinity and indicated that 95% of the terrestrial MFs were also detected with the δ^13^C and salinity approaches (Kong et al., submitted).^51^ Supporting that C475 is a representative proxy for terrestrial DOM.

### 4.5 Tuning Grid Decisions and Their Influence on Model Performance

The performance and the key features changed when the preprocessing and hyperparameters of the experiment changed. This is especially true for the tuning grid that is searched during the training of the model. Due to the computational cost, especially for the RFs, we decided not to use a nested cross-validation. We tried to counteract the possible bias by creating similar training and test sets. Repeated training of cross-validation hyperparameter searches for the high-error GLMs showed that the mean error did not vary much between preprocessing and machine learning method combinations, indicating a successful split of the training and test set. The two examples, on which repeated cross-validation was performed, showed results similar to the results from the heat maps, indicating that overfitting in these cases was not induced by the one-time split of the training and test data. While RFs can cope with high feature numbers and the GLM is regularized by an elastic net, the SVMs do not have such mechanics. The SVMs where overfitting occurred on the unfiltered data likely originated from a suboptimal choice of kernels. Our choice of polynomial and radial basis function kernels was driven by their widespread use and the ability to capture nonlinear relationships. A previous study showed that linear kernels perform better for cases where overfitting is a problem.^54^

One of the key aspects for RFs is the mtry parameter. A larger mtry value was suitable for data sets with large parameter counts. This led to more focused key features, as the few stronger predictors appeared more often in the selected subset.^55^ Limiting the mtry values to a smaller percentage of features may have been more suitable to find the more subtle features. With MS data, we therefore may not want to tune mtry to very high values to avoid reducing the importance of many less obvious features. A strategy for future work could be to perform model training with different mtry value limitations to compare the important features in the data. For generalized linear models, this could be tuned via the alpha parameter. With fewer feature weights being reduced to zero, the weights would be spread across the data. Instead of automated feature selection by the elastic net,^56^ selecting a fixed alpha value and tuning lambda may be beneficial as a feature selection method.

We also considered GLMs, since they are the fastest to tune and allow easy usage of the weights as importance measurement and automatic feature selection by changing the elastic net, which also helps against overfitting. This was mostly achieved with the exception of the ALR transformation, revealing the necessity to evaluate normalization methods. The repeated cross-validations also showed that in the cases of overfitting, there were no outliers and a stable performance in the case of SUM normalization was achieved. For the lasso regression in GLMs, all features should be on the same scale to avoid introducing a bias. This was not done by rescaling the features between the normalization and the model training step (preprocess in the package caret). The found key features were not the most intense features (Figure S5) as the selected weights are not from the features with the highest mean abundance.

## 5 Conclusions

Several state-of-the-art machine learning methods were applied in order to predict the PARAFAC component C475, which indicates the terrestrial contribution in DOM, based on a combination of molecular formulas and their retention time. The MF and RT measurements were obtained via modern LC-FTMS, which, most recently, can be applied directly to saltwater samples. Using our methods, we were able to predict our proxy with an NRMSE of only 5.7% of the original scale of the proxy. Moreover, the MFs that were predicted as terrestrial were different from those that were identified with prior methods, not taking into account the RT values. Our methods were thus able to produce new chemical insights showing trends similar to those in the literature while giving a guideline for ML-based approaches to DOM problems using LC-FTMS: for improved model performance, we generally suggest filtering the data at least for MFs with low variance. For normalization, we recommend not using the ALR normalization, and for time-aware/time-agnostic data, we found no preference. We suggest RFs for data that are not filtered at all or approaches that require nonlinear assumptions and GLM for faster computation times.

In the future, it would be desirable to investigate more closely the chemical characteristics of those MFs that are the best predictors for terrestrial DOM. Is it possible, using our methods, to further narrow the search for the actual molecules that are represented by the MFs? As it turns out, our methods are able to make good predictions using only around 2000 (as compared to the original over 70,000 features). It would be interesting to further investigate these 2000 important features. How can they be characterized? Is it possible to combine or reduce these features further to a much smaller set of features while still being able to make good predictions?

Can our models be applied to water samples stemming from other regions of the ocean, in particular, to regions that are far less terrestrial than the Arctic Ocean (e.g., the Antarctic Ocean)? It is an intriguing open question whether the identical set of important features will be applicable to such samples, which will be addressed in a follow-up study.

## Supplementary Material



## Data Availability

The used EEM data and PARAFAC components are available at https://doi.pangaea.de/10.1594/PANGAEA.948019. The LC-FTMS data are part of another publication and will be available under the following link when that publication is accepted: 10.6084/m9.figshare.29210642. The code can be made available upon request.

## Notes

Boxplot comparing the 500 RFs, three plots regarding the feature consistency in RFs over 1000 repeats, a barplot of the SHAP value distribution over the mass, and heatmaps for the *R* ^2^ and mean absolute error for low variance including and excluding experiments (PDF)
