# Supplementary material for: Optimizing Machine Learning-Based Prediction of Terrestrial Dissolved Organic Matter in the Ocean Using Fluorescence and LC-FTMS Data
Source: ACS Omega. 2025 Jul 3;10(27):29497–509. doi: 10.1021/acsomega.5c02849 (PMC12268369; doi:10.1021/acsomega.5c02849)
Supplement: Supplementary file 1 [file ao5c02849_si_001.pdf]

Optimizing machine learning-based prediction of terrestrial dissolved organic matter in the ocean using fluorescence and LC-FTMS data

Marlo Bareth,[*,†,‡] Boris P. Koch,[*,†,¶] Gabriel Zachmann,[‡] Xianyu Kong,[†] Oliver J. Lechtenfeld,[§] and Sebastian Maneth[‡]

†*Alfred-Wegener-Institut Helmholtz-Zentrum für Polar- und Meeresforschung, Ecological chemistry department, Am Handelshafen 12, 27570 Bremerhaven, Germany*

‡*University of Bremen, Faculty 3 – Mathematics and Computer Science, Bibliothekstr. 5, 28359 Bremen, Germany*

¶*University of applied sciences Bremerhaven, An d. Karlstadt 8, 27568 Bremerhaven, Germany*

§*Helmholtz Centre for Environmental Research – UFZ, Department Environmental Analytical Chemistry, Research group BioGeoOmics, Permoserstr. 15, 04318 Leipzig, Germany*

E-mail: Marlo.Bareth@awi.de; Boris.Koch@awi.de

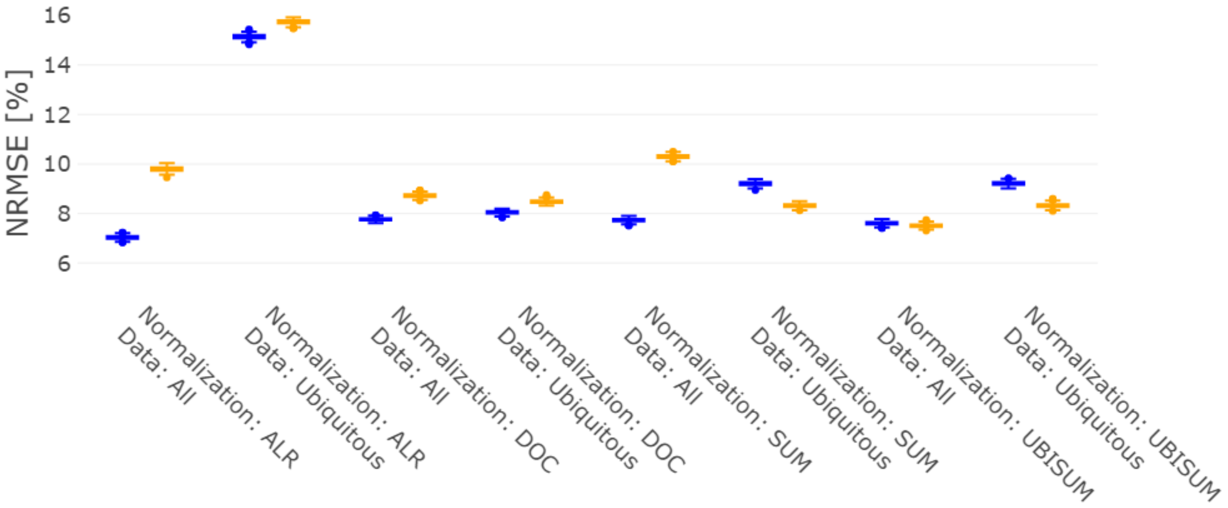

Figure S1: Uncertainty introduced by non-deterministic random forest models. Each box in the box plot represents the NRMSE for 500 runs of a random forest model experiment (based on all data). Time aware (feature: MFRTs, colored orange) and time agnostic data (features: molecular formula, blue) using different preprocessing combinations. Normalizations by DOC-content (DOC-N), sum of intensities in measurement (SUM), or sum of ubiquitous peaks over all samples (UBISUM). Additionally, additive log ratio transformation (ALR) based on the least varying ubiquitous feature was tested. Ubiquitous data was filtered to only include molecular formulas that were present in all samples.

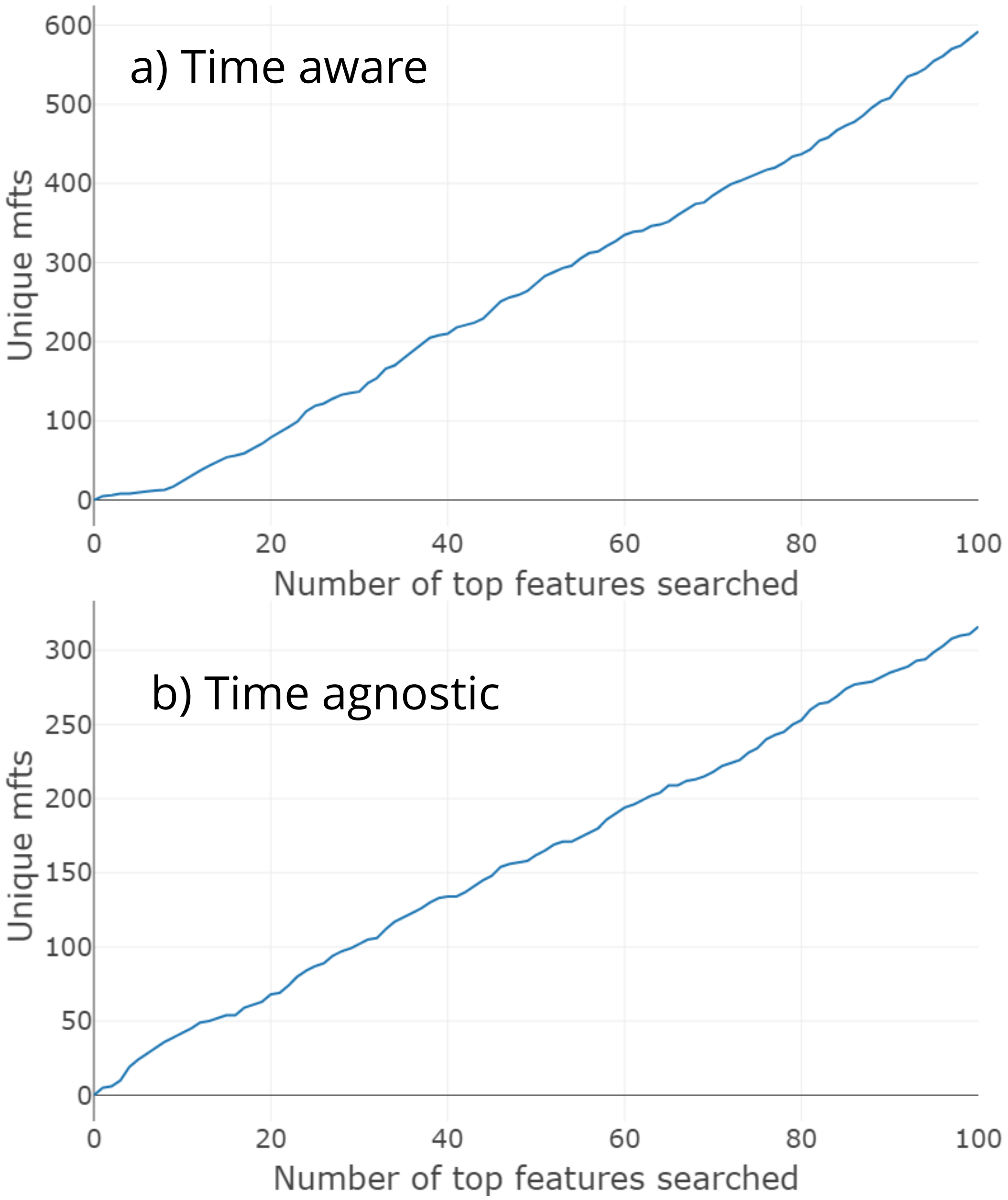

Figure S2: 1000 repeated runs of random forests (RF) with different seeds. When considering only a number of most important ranked features permutation importance (x-axis), showing the number of unique molecular formula time points (y-axis). Experiment was performed on the unfiltered (no ubiquitous filtering or excluding low variance), ubiquitous sum normalized, time aware (a) and time agnostic dataset (b).

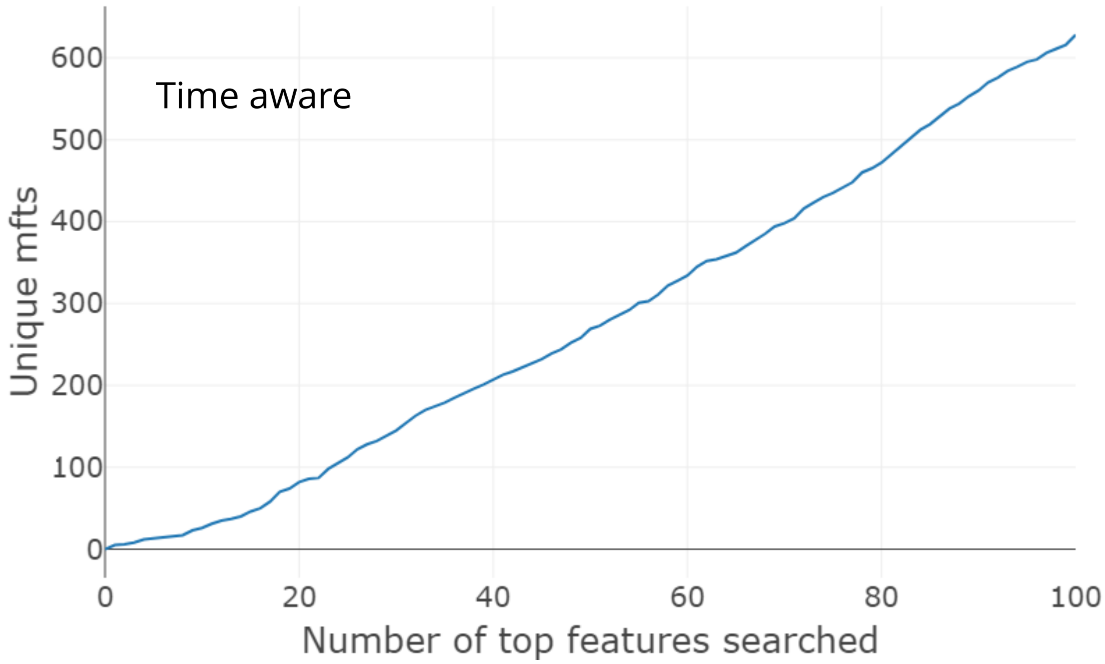

Figure S3: 1000 runs of random forests (RF) with different seeds based on time aware data. Only a number of most important ranked features based on SHAP values (x-axis) are considered, showing the resulting number of unique molecular time points. Experiment was performed on the unfiltered (no ubiquitous filtering or excluding low variance), ubiquitous sum normalized, time aware dataset.

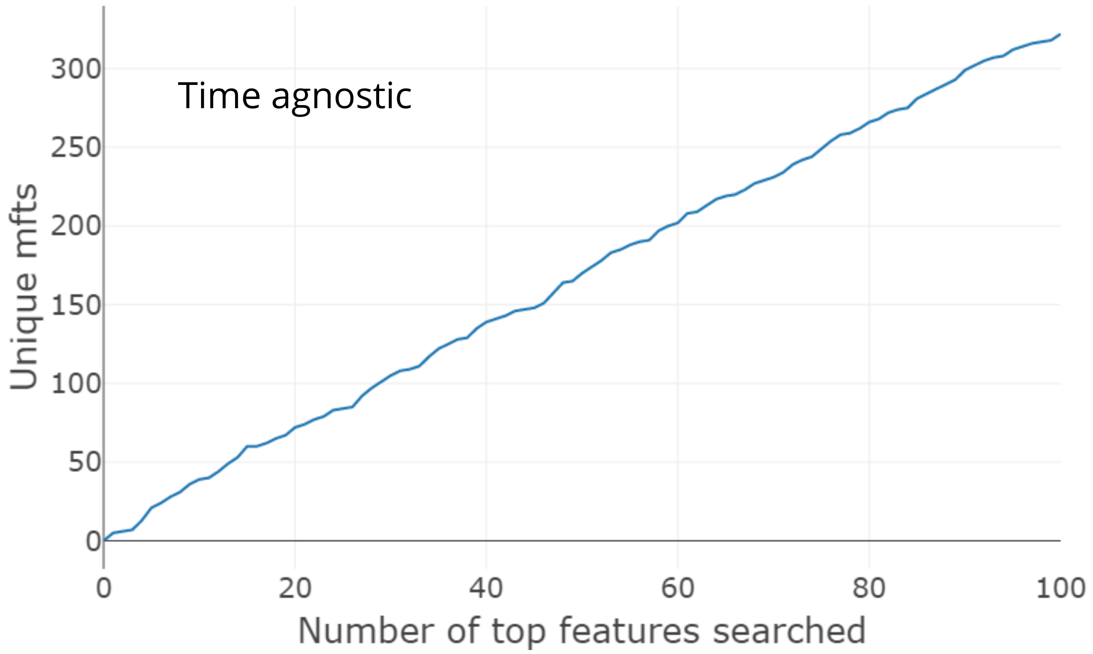

Figure S4: 1000 runs of random forests (RF) with different seeds based on time agnostic data. Only a number of most important ranked features based on SHAP values (x-axis) are considered, showing the resulting number of unique molecular time points. Experiment was performed on the unfiltered (no ubiquitous filtering or excluding low variance), ubiquitous sum normalized, time agnostic dataset.

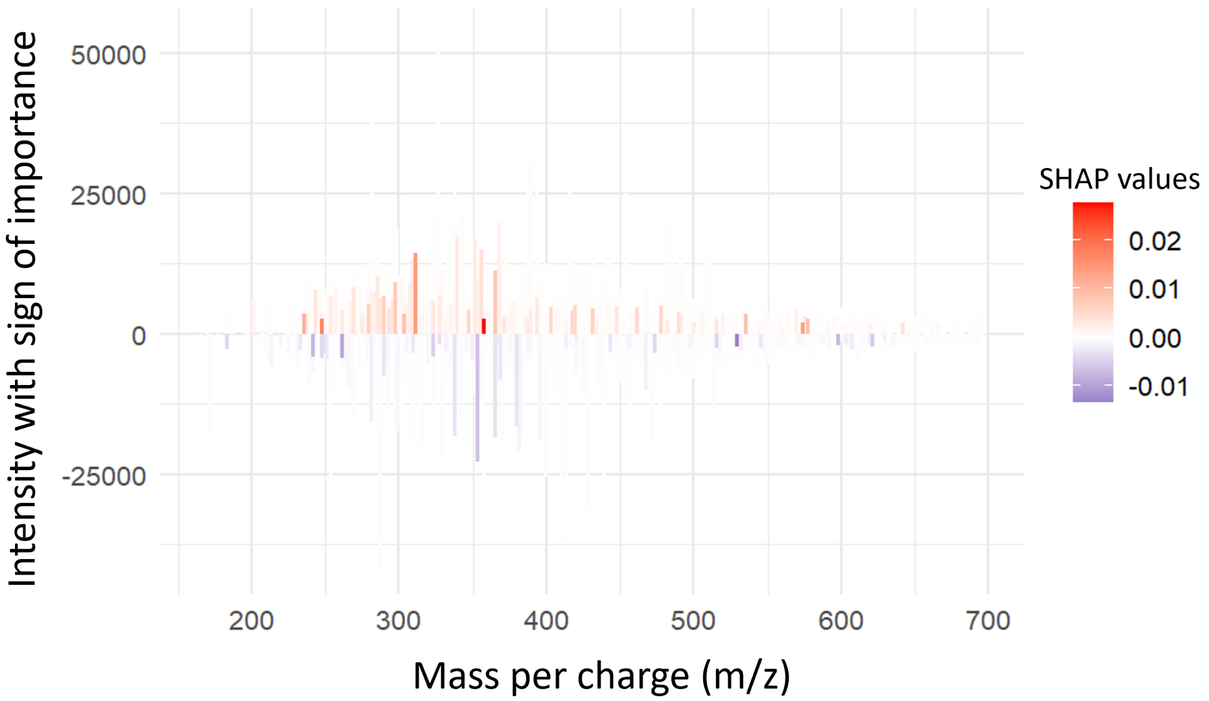

Figure S5: Mass spectrum of an average terrestrial sample showing SHAP values for the best performing random forest model. Positive SHAPs (red; important masses that were higher for samples with higher C475) and negative (blue; important masses that were lower for samples with higher C475) colored more intensely with absolute SHAP value magnitude. Model was based on log ratio transformed, including low variance data, time agnostic data.

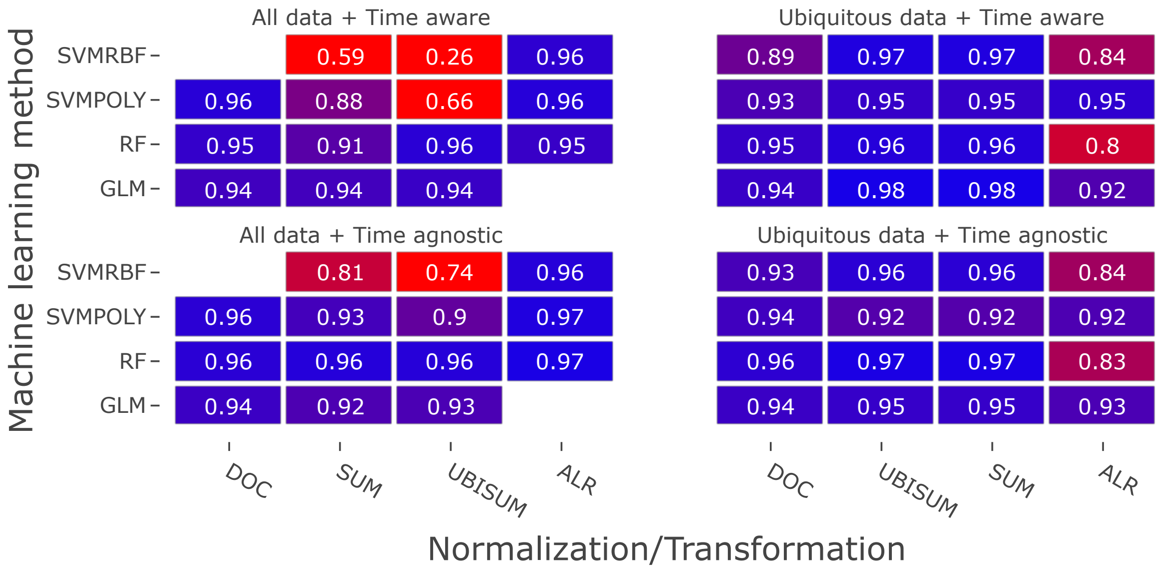

Figure S6: Model performance before removal of low variance features: The coefficient of determination R^2 as squared correlation of different preprocessing and machine learning model combinations using non variance filtered data predicting test set samples. Different normalizations (x axis) and different machine learning methods (y axis) per sub plot. Time aware data top row, time agnostic data bottom row. All features left column, only ubiquitous features right column. Results averaged for 500 Random Forest experiments each.

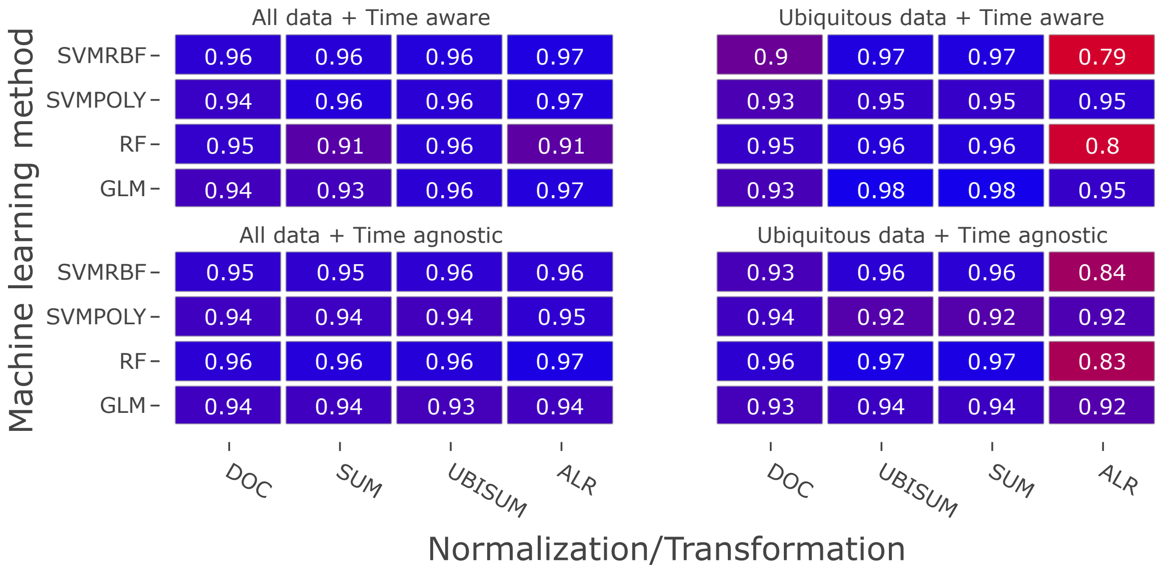

Figure S7: Model performance before removal of low variance features: The coefficient of determination R^2 as squared correlation of different preprocessing and machine learning model combinations using variance filtered data predicting the test set samples. Different normalizations (x axis) and different machine learning methods (y axis) per sub plot. Time aware data top row, time agnostic data bottom row. All features left column, only ubiquitous features right column. Results averaged for 500 Random Forest experiments each.

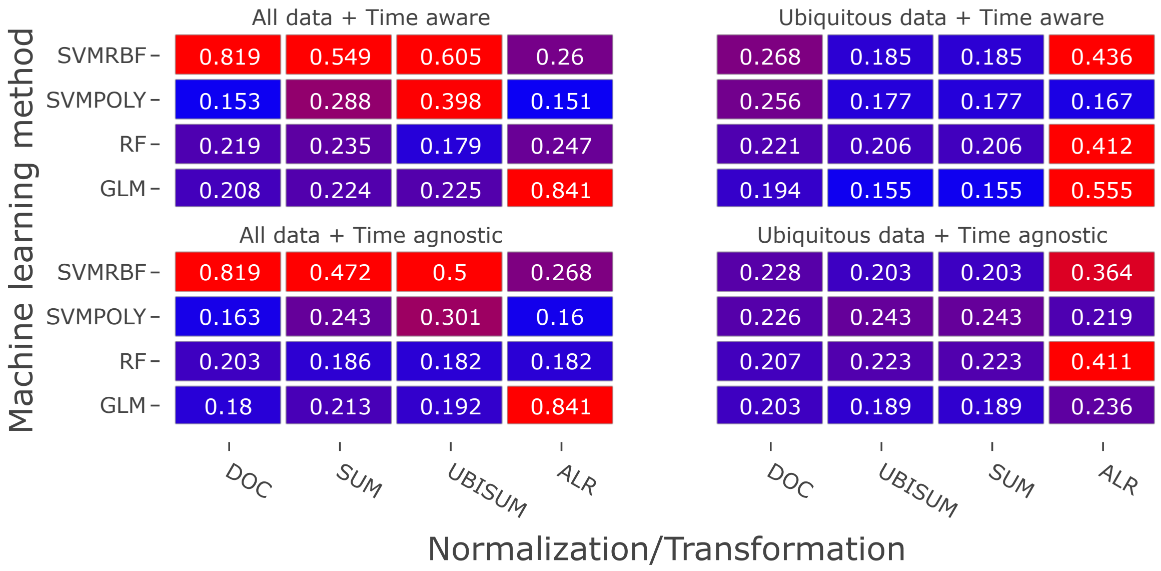

Figure S8: Model performance before removal of low variance features: The mean absolute error (MAE) for different tested combinations of non variance filtered data predicting the test set samples, scored in z-score normalized C475. Different normalizations (x axis) and different machine learning method (y axis) per sub plot. Time aware data top row, time agnostic data bottom row. All features left column, only ubiquitous features right column. Results averaged for 500 Random Forest experiments each.

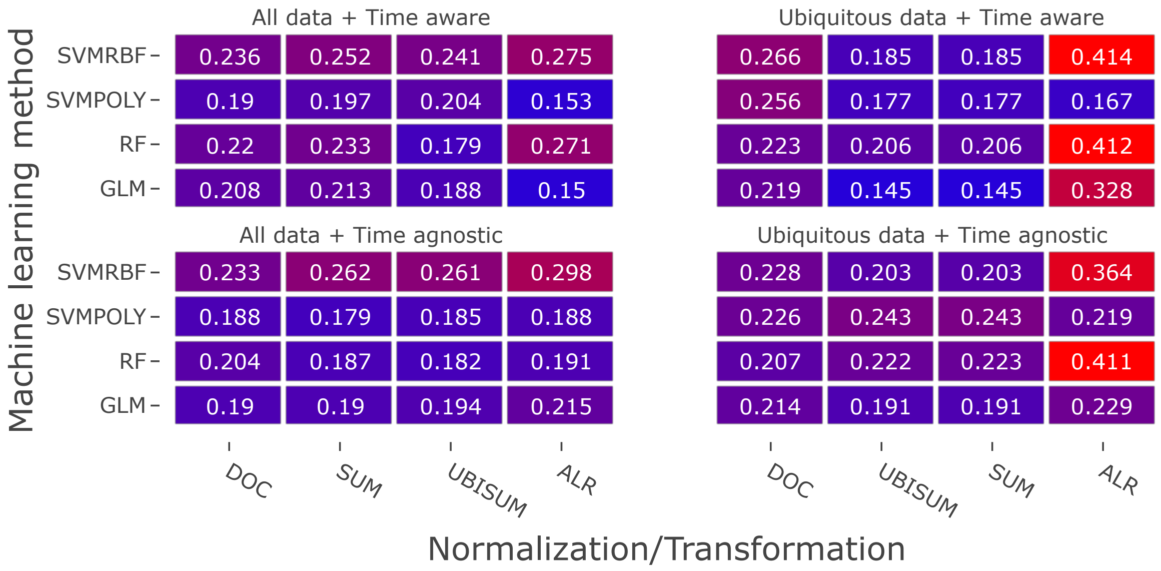

Figure S9: Model performance before removal of low variance features: The mean absolute error (MAE) for different tested combinations of variance filtered data predicting the test set samples, scored in z-score normalized C475. Different normalizations (x axis) and different machine learning method (y axis) per sub plot. Time aware data top row, time agnostic data bottom row. All features left column, only ubiquitous features right column. Results averaged for 500 Random Forest experiments each.
